# Withdrawal of sodium monensin when associated with virginiamycin during adaptation and finishing periods on feedlot performance, feeding behavior, carcass, rumen, and cecum morphometrics characteristics of Nellore cattle

**DOI:** 10.3389/fvets.2023.1067434

**Published:** 2023-01-24

**Authors:** André L. N. Rigueiro, Murilo C. S. Pereira, Antonio M. Silvestre, Ana Carolina J. Pinto, Luana D. Felizari, Evandro F. F. Dias, Breno L. Demartini, Daniela D. Estevam, João V. T. Dellaqua, Katia L. R. Souza, Leandro A. F. Silva, Ana B. P. C. Nunes, Johnny M. Souza, Danilo D. Millen

**Affiliations:** ^1^Department of Breeding and Animal Nutrition, School of Veterinary Medicine and Animal Science, São Paulo State University (UNESP), Botucatu, Brazil; ^2^Department of Animal Production, College of Agricultural and Technological Sciences, São Paulo State University (UNESP), Dracena, Brazil

**Keywords:** antibiotic, ionophore, papillae, rumen, Zebu

## Abstract

Feed additives such as monensin (MON) and virginiamycin (VM) are widely used in feedlots diets to maximize rumen fermentation. However, the knowledge about the effects of MON and VM combinations in specifics feedlot periods and the benefits of this association are still unclear. This study aimed to evaluate the effects of withdrawal of MON when associated with VM during the adaptation and finishing periods on feedlot performance of Nellore cattle. The experiment was designed as a completely randomized block replicated six times (four animals/pen) in which 120 Nellore bulls (378.4 ± 24.4 kg) were allocated in 30 pens and fed for 112 days according to the following treatments: (T1) MON during the entire feeding period; (T2) VM during the entire feeding period; (T3) MON+VM during the adaptation period and only VM during the finishing period 1 and 2; (T4) MON+VM during the entire feeding period; (T5) MON+VM during the adaptation and finishing period 1 and only VM during the finishing period 2. After 112 days on feed, no treatment effect was observed for DMI (*P* ≥ 0.12). However, bulls fed T5 had greater (*P* = 0.05) final BW and ADG when compared to T1, T2, and T4. Cattle from T3 and T5 groups presented heavier HCW (*P* = 0.05) than that fed T1, T2, and T4. Nellore bulls fed T1 and T5 had lower (*P* < 0.01) DMI variation than those receiving T2. The withdrawal of MON when associated with VM during the final third of the feedlot period improved overall final BW, ADG, and HCW when compared to bulls fed either MON or VM, but did not positively impact feedlot performance when compared to cattle that had MON withdrawn at the end of the adaptation period.

## 1. Introduction

Feed additives such as ionophores are widely used in North American and Brazilian feedlots (1, 2). In a meta-analysis conducted by Duffield et al. (3), sodium monensin (MON) decreased dry matter intake (DMI) by 3.1% and improved the gain-to-feed (G:F) ratio by 6.4% in feedlot cattle. Also, it has been reported by the Brazilian feedlot cattle nutritionists that the use of antibiotics associated with ionophores as a primary feed additive has become a common practice (2). The most common antibiotic that is associated with ionophores is virginiamycin (VM). Salinas-Chavira et al. (4) and Salinas-Chavira et al. (5) reported that VM improved G:F ratio by 3.83 and 4.20%, respectively when VM was fed to feedlot Holstein cattle. Both additives improved G:F ratio for feedlot cattle when fed as sole feed additives, however, only MON has been shown to decrease DMI (3).

There are few studies that evaluated the effect of MON and VM combinations for cattle, and the benefits of this association are still unclear. Erasmus et al. (6) reported a complementary effect between MON and VM when both were included in the diets of early lactation cows. However, Lemos et al. (7) did not observe any evidence that the combination of MON and VM for the entire feeding period improves feedlot performance or causes a positive impact on carcass characteristics of Zebu cattle. However, although the studies cited above have evaluated the combination between MON and VM in feedlot diets, none of them reported the combination of two feed additives in specifics periods. In the meantime, Rigueiro et al. (8) reported positive effects using different combinations of MON and VM in feedlot Nellore cattle. It was recommended by the authors that Nellore yearling bulls should be fed with diets containing MON and VM only during the adaptation period, and VM during the finishing period to improve overall feedlot performance. In a subsequent study, Rigueiro et al. (9) reported again that the use of MON and VM associated for the entire feeding period did not promote any positive effect on feedlot performance when compared to cattle fed only MON or VM. Furthermore, Rigueiro et al. (8, 9) observed that cattle fed only VM did not decrease DMI in the last 28 days on feed.

Typically, the DMI of feedlot cattle decreases in the final third of the feeding period, and this is one of the challenges feedlot cattle nutritionists have to face to keep cattle performance. One of the main factors related to DMI decrease during this period is the increase in leptin concentrations (10). According to Foote et al. (11), leptin was negatively associated with DMI. Leptin is a hormone produced by the adipocytes, and as an animal grows and approaches mature body size, fat deposition occurs as a normal part of growth (12). For this reason, nutritional strategies to increase DMI during the final third of the feedlot period have become a new research area.

Therefore, we aimed to test the hypothesis that withdrawing MON combined with a higher energy diet during the final third of the feedlot period increases DMI, and as a consequence improves feedlot performance and carcass traits of Nellore cattle. Thus, this study was designed to evaluate the effects of withdrawing MON when associated with VM during the adaptation and finishing periods on feedlot performance, feeding behavior, carcass, rumen, and cecum morphometrics characteristics of Nellore cattle.

## 2. Material and methods

All the procedures involving the use of animals in this study were in accordance with the guidelines established by the São Paulo State University Ethical Committee for Animal Research (protocol number CEUA 154/2016).

### 2.1. Animals and treatments

The trial was conducted at the São Paulo State University feedlot, Dracena campus, Brazil. One hundred and twenty 22-mo-old yearling Nellore bulls (378.44 ± 24.43 kg) were allocated in 30 pens (1.5 m of linear bunk space and 18 m^2^ of pen space per animal; *n* = 4 animals per pen) and fed for 112 days, according to the treatments: (1) MON during the entire feeding period (T1); (2) VM during the entire feeding period (T2); (3) MON + VM during the adaptation period and only VM during the finishing period 1 and 2 (T3); (4) MON + VM during the entire feeding period (T4); (5) MON + VM during the adaptation and finishing period 1 and only VM during the finishing period 2 (T5). Doses were based on Rigueiro et al. (8) when either MON (30 mg/kg of DM) or VM (25 mg/kg of DM) were fed as sole feed additives in the diet.

### 2.2. Feeding and management description

At the beginning of the study, all yearling bulls were dewormed (Ivermax, Dispec do Brasil, Maringá, BR). Cattle were fed *ad libitum* 3 times per day at 800 h (35% of total ration), 1,100 h (20% of total ration), and 1,600 h (45% of total ration), targeting 3 to 5% refusal with free-choice water access to a water trough. The experimental diets were formulated according to the Large Ruminant Nutrition System (LRNS; (13)) and are shown in Table 1. The step-up adaptation program consisted of *ad libitum* intake and lasted 14 days, where 3 adaptation diets containing 66, 72, and 78% concentrate were fed for 5, 4, and 5 days, respectively. The finishing period program also consisted of *ad libitum* intake and lasted 98 days, where 2 finishing diets containing 84%, and 88% concentrate were fed for 58, and 40 days, respectively.

**Table 1:** Feed ingredients and chemical composition of high-concentrate diets fed to Nellore yearling bulls (*n* = 30) during adaptation and finishing periods.

**Item**	**Percent of concentrate**				

	**66**	**72**	**78**	**84**	**88**
Days on feed, *n*	5	4	5	58	40
**Ingredients, % of DM** ^a^					
*Cynodon dactylon* hay	20.00	14.00	4.00	2.00	2.00
Sugarcane bagasse	14.00	14.00	18.00	14.00	10.00
Corn grain fine grind	46.00	54.00	62.00	70.00	76.70
Soybean meal	17.30	15.10	12.90	10.70	8.00
Supplement^b^	1.40	1.40	1.50	1.50	1.50
Urea	0.40	0.60	0.70	0.90	0.90
Limestone	0.90	0.90	0.90	0.90	0.90
**Nutrient content, % of DM** ^c^					
Dry matter, %	74.00	74.00	73.00	73.00	74.00
Total digestible nutrients	72.00	72.00	75.00	78.00	80.00
Crude protein	15.20	15.00	14.60	14.50	14.00
Neutral detergent fiber	34.30	30.50	26.80	23.00	19.20
Non-fiber carbohydrates	43.00	48.00	52.00	57.00	61.00
peNDF^d^	26.00	22.00	18.00	14.00	10.00
Net energy for gain, Mcal/kg	1.09	1.09	1.15	1.25	1.26
Ca	0.60	0.58	0.56	0.54	0.52
P	0.40	0.41	0.42	0.42	0.42

^a^DM: dry matter. ^b^Supplement contained: Ca: 18.23%; P: 4.05%, Mg: 0.77%, K: 0.05%, Na: 8.22%, Cl: 12.65%, S: 1.60%, Co: 27.50 ppm, Cu: 757.17 ppm, Fe: 2,498 ppm, I: 37.29 ppm, Mn: 740 ppm, Se: 6.20 ppm, Zn: 1,790 ppm. Monensin (Bovensin 200; Phibro Animal Health Corporation, Guarulhos, São Paulo, Brazil) was added at 2,000 mg/kg of supplement and Virginiamycin (V-Max 2; Phibro Animal Health Corporation, Guarulhos, São Paulo, Brazil) was added at 1,666 mg/kg of supplement and offered to yearling bulls in the treatments. ^c^Estimated by equations according to Large Ruminant Nutrition System (LRNS; (13)). ^d^peNDF: physically effective neutral detergent fiber.

Samples of the feed ingredients offered were analyzed for DM weekly and dietary DM was adjusted on a weekly basis according to changes in feed ingredient DM and water was added to the experimental diets to equalize the DM content by approximately 70%. Feed ingredient samples were dried in a forced-air oven for DM determination [(14); method 930.15]. Subsequently, samples were ground using a hammer mill to pass through a 1-mm screen (MA340, Marconi equipamentos para laboratórios Ltda, Piracicaba, BR) and were analyzed for ash [(14); method 942.05], crude protein [(15); method 990.02], and neutral detergent fiber (16).

### 2.3. Feedlot performance and carcass traits

One day before the start of the study, and every 28 days, all yearling bulls were withheld from feed for 16 h for the body weight (BW) assessment. Consequently, ADG and G:F ratio were calculated at the end of the experiment. The DMI was calculated daily by weighing the ration offered and refusal before the next morning delivery and expressed in kilograms and as a percentage of BW. The DMI variation was calculated as the difference in intake between two consecutive days throughout the study (17). Daily DMI variation was expressed as a percentage of variation. In order to estimate the net energy for maintenance (NEm) and net energy for gain (NEg), it was used the methods described by Lofgreen and Garrett (18), NRC (19), and Zinn and Shen (20).

Final BW was obtained at the feedlot prior to transportation. Cattle were transported 150 km (~3 h) to a commercial abattoir. Hot carcass weight (HCW) was obtained after a kidney, pelvic, and heart fat removal. Dressing percentage was calculated by dividing HCW by the final BW. The 12 th rib fat thickness, Biceps femoris fat thickness, longissimus muscle (LM) area, and marbling were measured *via* ultrasound at the beginning and at the end of the experimental period following the method described by Perkins et al. (21). The 12th rib fat thickness daily gain, Biceps femoris fat daily gain, and LM area daily gain were calculated as the difference between the two measurements divided by days on feed. Images were collected using an Aloka SSD-1100 Flexus RTU unit (Aloka Co. Ltd., Tokyo, Japan) with a 17.2 cm, 3.5 MHz probe.

### 2.4. Feeding behavior and particle sorting

All yearling bulls were submitted to visual observations to evaluate feeding behavior, every 5 min, over two periods of 24 h. The visual observations were performed on days 61 (finishing period 1) and 96 (finishing period 2) according to Robles et al. (22). Feeding behavior data were recorded for each animal as follows: time spent resting, ruminating, and eating (expressed in minutes), and the number of meals per day. A meal was considered the non-interrupted time cattle stayed in the feed bunk eating the ration. Meal length in minutes was calculated by dividing time spent eating by the number of meals per day. The DMI per meal in kilograms was calculated by dividing DMI by the number of meals per day.

In addition, data on time spent eating and ruminating were used to calculate the eating rate of DM (time spent eating/DMI) and rumination rate of DM (time spent ruminating/DMI), both expressed in minutes per kilogram of DM, according to Pereira et al. (23). Samples of diets and refusals were collected for chemical analysis of NDF (16) to determine the intake of NDF on the day of feeding behavior. Eating rate of NDF was calculated by dividing the time spent eating by NDF intake. Rumination rate of NDF was determined by dividing the time spent ruminating by NDF intake. Both eating rate and rumination rate were expressed in minutes per kilogram of NDF, according to Pereira et al. (23).

Samples of diets and refusals were also collected on days 61 and 62 (finishing period 1), and 96 and 97 (finishing period 2) of the study, respectively, for the determination of particle-size distribution using the Penn State Particle Separator with aperture sizes of 19, 8, and 1.18 mm, and a pan according to Heinrichs and Kononoff (24). The particle size distribution was determined using representative 1-L samples. Physical effectiveness factor was determined as the proportion of particles retained on 3 sieves (25).

Samples of diets and orts were also collected for the determination of particle size distribution, which was performed by sieving using the Penn State Particle Size Separator and reported on an as-fed basis as described by Heinrichs and Kononoff (24). Particle sorting was determined as follows: n intake / n predicted intake, in which *n* = particle fraction retained on screens of 19 mm (long), 8 mm (medium), and 1.18 mm (short) and a pan (fine). Particle sorting values equal to 1 indicate no sorting. Those < 1 indicate selective refusal (sorting against), and those >1 indicate preferential consumption (sorting for), according to Leonardi and Armentano (26).

### 2.5. Liver abscess, rumen and cecum morphometrics

Liver abscesses were classified according to incidence according to Brink et al. (27). Rumenitis evaluation was recorded after cattle evisceration, and all entire washed rumens were scored. Rumen epithelium was classified according to the incidence of lesions (rumenitis) and abnormalities (e.g., papillae clumped) as described by Bigham and McManus (28) using a scale of 0 (no lesions and abnormalities noted) to 10 (severe ulcerative lesions). All rumens were scored by 2 trained individuals, who were blinded to the treatments, and the final data represent the average of the 2 scores.

Also, a 1-cm^2^ fragment of each rumen was collected from the dorsal cranial sac and placed into a PBS solution for morphometric measurements according to Resende Júnior et al. (29). Manually, the number of papillae per square centimeter of rumen wall (NOP) was determined; 12 papillae were randomly collected from each fragment and scanned, and the mean papillae area (MPA) was determined using an image analysis system (Image Tool, version 2.01 alpha 4, UTHSCSA Dental Diagnostic Science, San Antonio, TX). The rumen wall absorptive surface area (ASA) in cm^2^ was calculated as follows: 1 + (NOP × MPA) – (NOP × 0.002), where 1 represents the 1 cm^2^ fragment collected and 0.002 is the estimated basal area of papillae in square centimeters. The papillae area, expressed as a percentage of ASA, was calculated as follows: (NOP × MPA)/ASA × 100.

Likewise, a 1-cm^2^ fragment of each rumen was collected from the ventral cranial sac for histological assessment. Histological sections were stained with hematoxylin and eosin, embedded in paraffin wax, and sectioned (30). Histological measurements, such as papillae height, papillae width, papillae surface area, and keratinized layer thickness were performed on 4 papillae per animal using a computer-aided light microscope image analysis. The same 1-cm^2^ fragment collected from the ventral cranial sac was also used for the evaluation of cell proliferation of rumen papillae according to the immunohistochemistry method adapted from Pereira et al. (31). The slides were incubated with primary inoculum (PCNA-PC10, Dako, Glostrup, Denmark), and diluted in PBS for positive control. For the negative control, only PBS was added to the IgG Murine Anti-Mouse Isotope Control (Sigma, Saint Louis, MO, EUA) in a dark moist refrigerated chamber (4°C) overnight with a 1:500 dilution, and utilizing 70 μL per sample (for both controls). The slides were then mounted for analyses using a Leica Qwin Image Analyzer within a Leica electron light microscope. Four papillae of each animal were randomly chosen (final data represented the average of the 4 papillae) to determine the number of cell nuclei, as well as the number of proliferating cell nuclei. The cell proliferation index was expressed as a percentage and was calculated as follows: number of proliferating nuclei cells/number of cell nuclei × 100.

Cecum lesions evaluation was performed after cattle evisceration, and all washed cecum were scored. Cecum epithelium was classified according to the presence of cecal wall inflammation, lesions, and petechiae using a scale of 0 (no lesions noted) to 10 (severe lesions), according to Pereira et al. (32). All cecum were scored by 2 trained individuals, who were blinded to the treatments, and the final data represented the average of the 2 scores. In addition, a 1 cm^2^ fragment was collected from the center of the cecum epithelium for histological assessment and preserved in buffered paraformaldehyde 4% solution until future histological analyses (33). For the histological analysis of cecum epithelium, tissue samples were dehydrated and embedded in paraffin wax, sectioned at 8 μm, and stained with hematoxylin and eosin. Histological measurements, such as crypt depth and goblet cells, were determined in 10% of the total number of crypts per animal, using a Leica Qwin Image Analyzer within a Leica electron light microscope.

### 2.6. Statistical analysis

The experimental design was a completely randomized block and the initial BW was utilized as a criterion for block formation, and the block was included in the model as a random effect. Pens were considered experimental unit for this study (*n* = 30; 4 bulls per pen), and each treatment was replicated 6 times (block 1: 338.42 kg; block 2: 358.90 kg; block 3: 371.70 kg; block 4: 386.50; block 5: 395.60 kg; block 6: 412.50 kg; SEM: 9.96). Data were analyzed using the MIXED procedure of SAS (SAS Inst., Inc., Cary, NC) and Tukey test to compare means. Tests for normality (Shapiro-Wilk and Kolmogorov-Smirnov) and heterogeneity of treatment variances (GROUP option of SAS) were performed before analyzing the data. Results were considered significant at *P* ≤ 0.05 level.

## 3. Results

### 3.1. Feedlot performance and carcass traits

Regarding the performance data, the initial BW was not affected by treatments (*P* = 0.99; Table 2). For the first 28 days on feed, there were no effects of treatments for final BW (*P* = 0.10), and DMI (*P* ≥ 0.35), expressed both in kg and as a percentage of BW. However, Nellore bulls fed T5 had greater (P ≤ 0.03) ADG and improved G:F ratio, when compared to animals receiving T2, T3, and T4. Cattle fed T2 had greater (*P* = 0.02) DMI variation than animals from other treatments. After 56 days on feed, no significant treatments effects were observed for final BW (*P* = 0.11), and DMI (*P* ≥ 0.49), expressed both in kg and as a percentage of BW. However, cattle fed T5 had greater (*P* = 0.05) ADG and improved G:F ratio when compared to animals receiving T1, T2, and T4. Cattle fed T1 had lower (*P* = 0.04) DMI variation than those receiving T2 and T4.

**Table 2:** Withdrawal of sodium monensin when associated with virginiamycin during adaptation and finishing periods on feedlot performance of Nellore yearling bulls (*n* = 30) consuming high-concentrate diets.

**Item^2^**	**Period**	**Treatments** ^**1**^					**s.e.m.^3^**	**P-value**

	**Adaptation:**	**MON**	**VM**	**MONVM**	**MONVM**	**MONVM**		
	**Finishing 1:**	**MON**	**VM**	**VM**	**MONVM**	**MONVM**		
	**Finishing 2:**	**MON**	**VM**	**VM**	**MONVM**	**VM**		
		**(T1)**	**(T2)**	**(T3)**	**(T4)**	**(T5)**		
Initial BW, kg		378.23	378.33	378.60	378.57	378.48	9.96	0.99
**0–28 days**								
Final BW, kg		411.44	402.68	409.52	409.57	417.60	10.75	0.10
ADG, kg		1.18^ab^	0.87^c^	1.10^bc^	1.11^bc^	1.40^a^	0.11	0.03
Daily DMI, kg		8.62	8.58	8.72	8.29	8.58	0.35	0.59
Daily DMI, % of BW		2.06	2.10	2.13	2.06	2.09	0.03	0.47
G:F ratio, kg/kg		0.140^ab^	0.102^c^	0.126^b^	0.131^b^	0.160^a^	0.011	0.01
DMI variation, %		7.29^b^	9.88^a^	7.43^b^	7.71^b^	7.11^b^	0.62	0.02
**0–56 days**								
Final BW, kg		448.84	444.56	455.20	448.79	459.64	12.17	0.11
ADG, kg		1.26^bc^	1.18^c^	1.36^ab^	1.25^bc^	1.45^a^	0.07	0.05
Daily DMI, kg		9.07	9.19	9.36	9.00	9.40	0.30	0.51
Daily DMI, % of BW		2.02	2.07	2.06	2.00	2.04	0.03	0.49
G:F ratio, kg/kg		0.139^bc^	0.128^c^	0.146^ab^	0.139^bc^	0.155^a^	0.006	0.03
DMI variation, %		5.96^c^	8.25^a^	7.04^abc^	7.56^ab^	6.10^bc^	0.57	0.04
**0–72 days**								
Final BW, kg		472.72^bc^	468.24^c^	479.84^ab^	473.00^bc^	485.80^a^	11.50	0.04
ADG, kg		1.31^bc^	1.24^c^	1.40^ab^	1.30^bc^	1.49^a^	0.05	0.02
Daily DMI, kg		9.31	9.46	9.67	9.19	9.62	0.30	0.39
Daily DMI, % of BW		1.97	2.02	2.02	1.94	1.98	0.03	0.29
G:F ratio, kg/kg		0.141^bc^	0.132^c^	0.145^b^	0143^b^	0.156^a^	0.005	< 0.01
DMI variation, %		5.48^b^	7.57^a^	6.67^ab^	7.39^a^	5.92^b^	0.46	0.01
**0–84 days**								
Final BW, kg		487.40^bc^	485.36^c^	496.96^ab^	488.31^bc^	501.28^a^	12.23	0.05
ADG, kg		1.29^*bc*^	1.27^c^	1.40^ab^	1.30^bc^	1.46^a^	0.05	0.02
Daily DMI, kg		9.40	9.55	9.79	9.24	9.71	0.30	0.31
Daily DMI, % of BW		1.92	1.97	1.97	1.89	1.94	0.03	0.30
G:F ratio, kg/kg		0.139^bc^	0.133^c^	0.144^ab^	0.142^bc^	0.152^a^	0.005	0.03
DMI variation, %		5.49^b^	7.55^a^	6.61^ab^	7.23^a^	5.73^b^	0.44	< 0.01
**0–112 days**								
Final BW, kg		533.67^bc^	529.25^c^	540.79^ab^	531.18^bc^	548.25^a^	13.43	0.05
ADG, kg		1.39^b^	1.34^b^	1.45^ab^	1.36^b^	1.51^a^	0.05	0.05
Daily DMI, kg		9.50	9.69	9.97	9.27	9.94	0.31	0.14
Daily DMI, % of BW		1.78	1.83	1.83	1.74	1.81	0.03	0.12
G:F ratio, kg/kg		0.146^ab^	0.140^b^	0.145^ab^	0.147^ab^	0.153^a^	0.004	0.05
DMI variation, %		5.26^b^	7.13^a^	6.93^a^	6.74^a^	5.46^b^	0.41	< 0.01
NEm (Mcal/kg of DM)		2.03	1.96	2.00	2.04	2.08	0.03	0.11
NEg (Mcal/kg of DM)		1.37	1.31	1.34	1.39	1.41	0.03	0.11

^1^T1 (MON during the entire feeding period); T2 (VM during the entire feeding period); T3 (MON + VM during the adaptation period and only VM during the finishing period 1 and 2); T4 (MON + VM during the entire feeding period); T5 (MON + VM during the adaptation and finishing period 1 and only VM during the finishing period 2). ^2^BW, body weight; G: F, gain-to-feed ratio; AGD, average daily gain; DMI, dry matter intake; NEm, energy for maintenance; NEg, net energy for gain. ^3^s.e.m.: standard error of the mean, referent to *n* = 6 pens per treatment. Values within a row with different lower case letters differ (*P* < 0.05).

After 72 days on feed, no significant treatments effects were observed for DMI (*P* ≥ 0.29; Table 2), expressed both in kg and as a percentage of BW. However, cattle fed T5 had greater (*P* = 0.04) final BW when compared to animals receiving T1, T2, and T4. Nellore bulls fed T5 presented grater (*P* = 0.02) ADG than those receiving T2. Cattle fed T5 improved (*P* < 0.01) G:F ratio when compared to animals from other treatments. Nellore bulls fed T1 and T5 had lower (*P* = 0.01) DMI variation than those receiving T2 and T4.

After 84 days on feed, no significant treatments effects were observed for DMI (*P* ≥ 0.30; Table 2), expressed both in kg and as a percentage of BW. However, Nellore bulls fed T5 had greater (*P* < 0.05) final BW and ADG when compared to animals receiving T1, T2, and T4. Cattle fed T5 improved (*P* = 0.03) G:F ratio when compared to animals receiving T1, T2, and T4. Nellore bulls fed T1 and T5 had lower (*P* < 0.01) DMI variation than animals receiving T2 and T4.

Overall, after 112 days on feed, no significant treatments effects were observed for DMI (*P* ≥ 0.12; Table 2), expressed both in kg and as a percentage of BW, NEm (*P* = 0.11) and NEg (*P* = 0.11). However, bulls fed T5 had greater (*P* = 0.05) final BW and improved G:F when compared to animals receiving T2. Cattle fed T5 had greater (*P* = 0.05) ADG when compared to animals receiving T1, T2, and T4. Nellore bulls fed T1 and T5 had lower (*P* < 0.01) DMI variation than animals from other treatments.

There was no significant treatment effect (*P* > 0.05) for most of the carcass characteristics variables evaluated (Table 3). However, cattle fed T3 and T5 had heavier (*P* = 0.05) HCW when compared to other treatments. Nellore bulls fed T3, T4, and T5 increased (*P* < 0.01) final 12th rib fat and 12th rib fat daily gain when compared to other treatments. Cattle fed T3 increased (*P* < 0.01) final BF fat thickness when compared to animals receiving T1 and T2. Moreover, cattle fed T1 reduced (*P* < 0.01) BF fat daily gain when compared to others treatments.

**Table 3:** Withdrawal of sodium monensin when associated with virginiamycin during adaptation and finishing periods on carcass characteristics of Nellore yearling bulls (*n* = 30) consuming high-concentrate diets.

**Item^2^**	**Period**	**Treatments** ^**1**^					**s.e.m.^3^**	**P-value**

	**Adaptation:**	**MON**	**VM**	**MONVM**	**MONVM**	**MONVM**		
	**Finishing 1:**	**MON**	**VM**	**VM**	**MONVM**	**MONVM**		
	**Finishing 2:**	**MON**	**VM**	**VM**	**MONVM**	**VM**		
		**(T1)**	**(T2)**	**(T3)**	**(T4)**	**(T5)**		
Hot carcass weight, kg		289.48^b^	289.02^b^	295,06^a^	289.12^b^	296,71^a^	7.97	0.05
Dressing percentage		54.25	54.61	54.58	54.39	54.09	0.31	0.47
Initial 12^th^ rib fat, mm		2.29	2.31	2.40	2.40	2.42	0.07	0.50
Final 12^th^ rib fat, mm		5.03^b^	5.26^b^	5.80^a^	5.94^a^	5.75^a^	0.20	< 0.01
12^th^ rib fat daily gain, mm		0.024^b^	0.025^b^	0.030^a^	0.031^a^	0.029^a^	0.001	< 0.01
Initial BF^2^ fat thickness, mm		4.16	4.12	4.09	4.01	4.15	0.10	0.84
Final BF fat thickness, mm		7.55^c^	8.36^b^	9.00^a^	8.50^ab^	8.57^ab^	0.24	< 0.01
BF fat daily gain, mm		0.030^c^	0.037^b^	0.043^a^	0.040^ab^	0.039^ab^	0.002	< 0.01
Initial LM^3^ area, cm^2^		63.19	61.04	60.73	62.99	59.89	1.84	0.14
Final LM area, cm^2^		79.81	77.72	77.92	77.39	78.65	1.93	0.86
LM area daily gain, cm^2^		0.150	0.150	0.150	0.140	0.163	0.01	0.79
Initial marbling, %		2.06	2.01	2.14	2.27	2.19	0.12	0.48
Final marbling, %		2.82	2.70	2.73	2.92	2.86	0.08	0.23

^1^T1 (MON during the entire feeding period); T2 (VM during the entire feeding period); T3 (MON + VM during the adaptation period and only VM during the finishing period 1 and 2); T4 (MON + VM during the entire feeding period); T5 (MON + VM during the adaptation and finishing period 1 and only VM during the finishing period 2). ^2^BF, *Biceps femoris* muscle; LM, Longissimus muscle. ^3^s.e.m., standard error of the mean, referent to *n* = 6 pens per treatment. Values within a row with different superscripts differ (*P* < 0.05).

### 3.2. Feeding behavior and particle sorting

No significant treatment effect was observed (*P* > 0.05) for most of the feeding behavior and particle sorting variables evaluated after 61 days on feed (Table 4). However, Nellore bulls fed T4 spent more time resting (*P* = 0.05) when compared to other treatments. Animals fed T3 and T5 sorted more intensively for long particles when compared to animals receiving T1 and T4. In addition, cattle fed T4 and T5 sorted (*P* = 0.05) against fine particles when compared to animals receiving T1 and T2.

**Table 4:** Withdrawal of sodium monensin when associated with virginiamycin during adaptation and finishing periods on feeding behavior and particle sorting at day 61 (finishing period 1) of Nellore yearling bulls (*n* = 30) consuming high-concentrate diets.

**Item^2^**	**Period**	**Treatments** ^**1**^					**s.e.m.^3^**	**P-value**

	**Adaptation:**	**MON**	**VM**	**MONVM**	**MONVM**	**MONVM**		
	**Finishing 1:**	**MON**	**VM**	**VM**	**MONVM**	**MONVM**		
	**Finishing 2:**	**MON**	**VM**	**VM**	**MONVM**	**VM**		
		**(T1)**	**(T2)**	**(T3)**	**(T4)**	**(T5)**		
**Feeding behavior**								
Time spent resting, min		994.17^b^	1002.08^b^	985.00^b^	1051.74^a^	980.63^b^	19.63	0.05
Time spent ruminating, min		292.50	274.17	272.71	220.14	289.37	21.87	0.16
Time spent eating, min		153.33	163.75	182.29	168.13	170.00	11.68	0.53
Meals per day, *n*		10.54	11.96	13.58	12.55	11.75	1.13	0.43
Meal length, min		14.91	14.04	13.62	13.69	15.12	1.05	0.70
DMI per meal, kg		0.96	0.88	0.76	0.83	0.92	0.09	0.55
DMI, kg		9.88	9.81	10.04	9.69	9.98	0.39	0.96
ER of DM^3^, min/kg of DM		15.72	17.06	18.17	17.74	17.05	1.48	0.91
RR of DM, min/kg of DM		30.11	28.04	27.00	23.20	29.15	2.32	0.26
NDF intake, kg		1.80	1.54	1.93	1.72	1.85	0.21	0.53
ER of NDF, min/kg of NDF		87.62	112.04	96.70	113.87	106.30	17.76	0.38
RR of NDF, min/kg of NDF		179.26	190.06	148.77	137.61	157.27	18.30	0.20
**Particle sorting** ^4^								
Long		0.91^b^	0.97^ab^	1.04^a^	0.92^b^	1.01^a^	0.06	0.05
Medium		0.98	0.95	0.99	1.06	1.03	0.03	0.15
Short		1.00	1.01	1.01	1.01	1.01	0.01	0.25
Fine		1.00^a^	1.00^a^	0.99^ab^	0.97^b^	0.97^b^	0.01	0.05

^1^T1 (MON during the entire feeding period); T2 (VM during the entire feeding period); T3 (MON + VM during the adaptation period and only VM during the finishing period 1 and 2); T4 (MON + VM during the entire feeding period); T5 (MON + VM during the adaptation and finishing period 1 and only VM during the finishing period 2). ^2^DMI, dry matter intake; ER, eating rate; DM, dry matter; RR, rumination rate; NDF, neutral detergent fiber; peNDF, physically effective neutral detergent fiber. ^3^s.e.m., standard error of the mean, referent to *n* = 6 pens per treatment. Values within a row with different lower case letters differ (*P* < 0.05). ^4^Particle fraction retained on screens of 19 mm (long), 8 mm (medium), 1.18 mm (short) and a pan (fine).

Also, no significant treatment effects were observed (*P* > 0.05) for most of the feeding behavior and particle sorting variables evaluated after 96 days on feed (Table 5). However, Nellore bulls fed T1 spent more time resting (*P* = 0.01) when compared to animals receiving T2 and T3. Cattle fed T2, T3, and T5 had greater (*P* = 0.04) DMI when compared to animals receiving T4.

**Table 5:** Withdrawal of sodium monensin when associated with virginiamycin during adaptation and finishing periods on feeding behavior and particle sorting at day 96 (finishing period 2) of Nellore yearling bulls (*n* = 30) consuming high-concentrate diets.

**Item^2^**	**Period**	**Treatments** ^**1**^					**s.e.m.^3^**	**P-value**

	**Adaptation:**	**MON**	**VM**	**MONVM**	**MONVM**	**MONVM**		
	**Finishing 1:**	**MON**	**VM**	**VM**	**MONVM**	**MONVM**		
	**Finishing 2:**	**MON**	**VM**	**VM**	**MONVM**	**VM**		
		**(T1)**	**(T2)**	**(T3)**	**(T4)**	**(T5)**		
**Feeding behavior**								
Time spent resting, min		1,068.67^a^	1,030.96^b^	1,015.62^b^	1,054.74^ab^	1,050.50^ab^	20.27	0.01
Time spent ruminating, min		210.13	247.84	245.00	219.38	225.88	13.47	0.19
Time spent eating, min		154.38	154.38	179.38	172.71	171.46	10.06	0.30
Meals per day, *n*		10.21	9.92	11.42	9.81	10.42	0.98	0.49
Meal length, min		16.52	15.88	15.91	18.34	17.10	1.69	0.83
DMI per meal, kg		1.06	1.08	0.93	1.04	1.07	0.12	0.87
DMI, kg		9.94^ab^	10.15^a^	10.51^a^	9.26^b^	10.72^a^	0.48	0.04
ER of DM^3^, min/kg of DM		15.78	15.61	17.18	19.11	16.06	1.42	0.39
RR of DM, min/kg of DM		21.51	24.57	23.52	23.87	21.15	1.44	0.26
NDF intake, kg		1.82	2.09	2.13	1.84	2.05	0.16	0.31
ER of NDF, min/kg of NDF		93.11	77.38	87.26	97.62	86.83	10.69	0.65
RR of NDF, min/kg of NDF		126.21	121.28	120.40	121.56	115.03	13.08	0.98
**Particle sorting** ^4^								
Long		1.06	1.08	1.06	1.01	1.03	0.02	0.26
Medium		1.02	1.03	1.03	1.01	1.03	0.01	0.85
Short		1.01	1.01	1.00	1.00	1.00	0.002	0.48
Fine		0.98	0.98	0.99	0.99	0.99	0.01	0.44

^1^T1 (MON during the entire feeding period); T2 (VM during the entire feeding period); T3 (MON + VM during the adaptation period and only VM during the finishing period 1 and 2); T4 (MON + VM during the entire feeding period); T5 (MON + VM during the adaptation and finishing period 1 and only VM during the finishing period 2). ^2^DMI, dry matter intake; ER, eating rate; DM, dry matter; RR, rumination rate; NDF, neutral detergent fiber; peNDF, physically effective neutral detergent fiber. ^3^s.e.m., standard error of the mean, referent to *n* = 6 pens per treatment. Values within a row with different lower case letters differ (*P* < 0.05). ^4^Particle fraction retained on screens of 19 mm (long), 8 mm (medium), 1.18 mm (short) and a pan (fine).

### 3.3. Liver abscess, rumen and cecum morphometrics

No liver abscess was found in the cattle evaluated in this study. No treatment effect was observed (*P* > 0.05) for any of the microscopic and cecum variables evaluated (Table 6). However, Nellore bulls fed T4 presented higher rumenitis scores (*P* = 0.05) when compared to animals receiving T2, T3, and T5. No treatment effect (*P* > 0.05) was observed for any of the macroscopic variables evaluated, except for mean papillae area (MPA), where cattle fed T1 and T4 had larger (*P* < 0.01) MPA when compared to other treatments.

**Table 6:** Withdrawal of sodium monensin when associated with virginiamycin during adaptation and finishing periods on rumen and cecum morphometrics of Nellore yearling bulls (*n* = 30) consuming high-concentrate diets.

**Item**	**Period**	**Treatments** ^**1**^					**s.e.m.^3^**	**P-value**

	**Adaptation:**	**MON**	**VM**	**MONVM**	**MONVM**	**MONVM**		
	**Finishing 1:**	**MON**	**VM**	**VM**	**MONVM**	**MONVM**		
	**Finishing 2:**	**MON**	**VM**	**VM**	**MONVM**	**VM**		
		**(T1)**	**(T2)**	**(T3)**	**(T4)**	**(T5)**		
**Rumen measurements**								
Rumenitis score		1.28^ab^	0.99^b^	1.08^b^	1.44^a^	1.01^b^	0.12	0.05
**Macroscopic variables**								
Number of papillae, *n*		75.24	79.48	79.56	72.74	81.35	6.53	0.84
Mean papillae area, cm^2^		0.54^a^	0.45^b^	0.42^b^	0.57^a^	0.43^b^	0.05	< 0.01
ASA^2^, cm^2^/cm^2^ of rumen wall		38.10	34.47	34.19	41.49	35.05	2.93	0.12
Papillae area, % of ASA		97.31	97.15	96.57	97.57	97.19	0.32	0.26
**Microscopic variables**								
Papillae height, mm		4.40	4.27	4.30	4.50	4.53	0.28	0.89
Papillae width, mm		0.46	0.40	0.44	0.43	0.44	0.02	0.08
Papillae surface area, mm^2^		1.83	1.59	1.80	1.77	1.84	0.12	0.42
Keratinized layer thickness, μm		12.33	11.86	11.70	11.60	11.91	0.40	0.78
Mitotic index, %		2.49	2.22	2.43	2.56	2.30	0.15	0.52
Mitotic index, *n*		49.71	44.33	48.67	51.13	46.08	3.15	0.52
**Cecum variables**								
Cecum score		2.08	2.21	2.29	1.99	1.79	0.42	0.92
Crypt depth, μm		170.63	154.23	150.65	162.77	162.77	11.20	0.42
Goblet cells, *n*		34.22	37.47	34.84	34.18	34.15	2.00	0.73

^1^T1 (MON during the entire feeding period); T2 (VM during the entire feeding period); T3 (MON + VM during the adaptation period and only VM during the finishing period 1 and 2); T4 (MON + VM during the entire feeding period); T5 (MON + VM during the adaptation and finishing period 1 and only VM during the finishing period 2). ^2^ASA, absorptive surface area. ^3^s.e.m., standard error of the mean, referent to *n* = 6 pens per treatment. Values within a row with different lower case letters differ (*P* < 0.05).

## 4. Discussion

The effect of combinations between MON and VM for cattle are unclear. Nuñez et al. (34) and Lemos et al. (7) tested combination of MON, lasalocid, VM, and flavomycin, but no positive feedlot performance effects were reported by the authors. Although the studies cited above have evaluated the combination between MON and VM in feedlot diets, none of them reported the combination of two feed additives in specifics periods. In this context, the present study was part of a larger research performed by this research group assessing the effect of different combinations of MON and VM in specifics feedlot periods. Rigueiro et al. (8) investigated different combinations of MON and VM during adaptation and finishing periods of feedlot Nellore cattle. The authors concluded that Nellore yearling bulls should be fed high-concentrate diets containing MON and VM during the adaptation period, and only VM during the finishing period to improve overall feedlot performance. Same authors also reported that cattle fed only VM during the finishing period had most of the increased performance in the last 40 days of the study. Consequently, it was hypothesized that the withdrawal of MON, when associated with VM, combined with a higher energy diet during the final third of the feedlot period would increase DMI, in order to improve feedlot performance and carcass traits.

In this context, DMI is an important indicator to evaluate how well cattle are either accepting or adapted to the diets (35), and the faster cattle reach a DMI of 2% of BW, more adapted they are to the diets. In the present study, there were no effects of treatments on DMI, expressed both in kg and as a percentage of BW, in the first 28 days on feed. Based on results described by Rigueiro et al. (8), where Nellore bulls fed VM as the sole feed additive during adaptation reached a DMI of 2% of the initial BW in 4.3 days on average, whereas those fed MON needed 20.7 days to reach a similar intake, another study of this research group was developed by Rigueiro et al. (9) where it was hypothesized that the adaptation period could be shortened to 9 days or even 6 days when VM (25 mg/kg DM) is used in finishing diets of Nellore cattle as the sole feed additive. The authors reported that, during the first 28 days on feed, the DMI decreased linearly as the adaptation was shortened for the cattle fed VM as a sole feed additive, where cattle fed VM for 14 days presented a greater DMI, expressed as % of BW, than animals fed either MON or MON+VM for 14 days.

The different combinations between MON and VM during adaptation and finishing periods in the current study did not affect the DMI expressed both in kg and as a percentage of BW overall. However, the MON effect on reducing DMI has been reported in the literature (3, 36, 37). In relation to the VM effect on DMI, Erasmus et al. (6), Lemos et al. (7) and Salinas-Chavira et al. (5) did not report a decrease in DMI when VM was fed to feedlot cattle. In the present study, Nellore bulls fed T5 did not consume MON during the final third of feedlot period (last 40 days), and therefore, DMI was not increased when compared to cattle fed MON + VM during the entire feeding period, which did not confirm one the hypothesis of the present study. On the other hand, Rigueiro et al. (8) observed an increase in DMI when MON was withdrawn during the finishing period. However, the finishing period lasted 71 days, 31 more days on feed when compared with the current study. Despite to the time of exposure to the treatment, the withdrawal of MON when associated with VM during the last 40 days of the feedlot period improved the animal performance overall when compared to bulls fed either MON or VM.

Although withdrawal of MON during the final third of the feedlot period does not affect DMI, cattle fed T5 consumed more 0.44 and 0.25 kg of DM per day when compared to cattle fed only MON and VM during the entire feeding period, respectively, which may have contributed to an increase HCW in 7.23 and 7.69 kg when compared to cattle fed only MON and VM, respectively. Nellore bulls fed only MON during the entire feeding period presented lower fat deposition. Goodrich et al. (36) also observed negative effects of MON on dressing percentage and 12th rib fat thickness. According to the authors, standard deviations for percentage change in carcass characteristics indicate that these effects of MON are highly variable. In a meta-analysis, Duffield et al. (3) did not report negative effects of feeding MON on carcass characteristics. However, the slower 12th rib fat daily gain and Biceps femoris fat daily gain observed in the current study, which led to thinner final 12th rib fat and final Biceps femoris fat thickness, may be associated with the decreased acetate:propionate ratio (38), which may negatively impact the lipid metabolism. It is noteworthy to mention that based on the results reported by Rigueiro et al. (9), the shortening of the adaptation period from 14 to 9 or 6 days did not negatively impact the feedlot performance overall, where there were no effects of treatments on final body weight, ADG and HCW. However, the authors reported that cattle fed VM for 9 days had more meals per day and less DMI intake per meal, due to the rumen acidification, resulting in a linear decrease in the 12th rib fat and BF fat daily gain.

Associated with the performance assay performed by Rigueiro et al. (9), Squizatti et al. (39) performed a rumen metabolism assay, and the authors reported that as the adaptation length decreased for animals consuming only VM, the rumen degradability of DM, NDF and starch decreased. Associated with that, it was observed higher proportions of protozoa for animals receiving VM adapted for 6 or even 9 days, justifying the reduced rate of ruminal degradation since protozoa predate bacteria, resulting in a reduction of colonization rate of feed and, consequently, reducing DMI as shown by Rigueiro et al. (9). In addition, both authors concluded that feedlot cattle fed VM as a sole feed additive should not be adapted to high-concentrate diets in less than 14 days, since it compromises DMI, carcass fat deposition and disrupts feeding behavior patterns.

In this context, analyzing the feeding behavior allows the adjustment of dietary management in order to achieve better production performance in beef cattle (40). The particle sorting affects the individual nutrient intake, since there are indications that this sorting of the diet is associated with an increased risk of metabolic diseases (41). In the present study, Nellore bulls fed T4 at day 61 sorted against long and fine particles, which led to spend more time resting. In addition, cattle fed T3 and T5 sorted in favor of long particle and against fine particles at day 61, which lead to a numerical increase in NDF intake and results in less time resting, which may be a response to control rumen acidification. Similarly, Rigueiro et al. (9) reported that the cattle adapted with VM for 9 days sorted for medium and against fine diet particles during both the adaptation and finishing periods and consumed significantly more NDF during adaptation.

It is recognized that reducing the particle size of fiber decreases chewing activity, saliva flow, and rumen pH, increasing the risk of subacute ruminal acidosis, as well as increasing resting time and reducing sorting behaviors and eating time (42). Consequently, increasing the consumption of large particles led to increased intake of physically effective fiber, which was positively associated with fiber digestion and chewing time, preventing subclinical ruminal acidosis (43). In relation to rumen measurements, rumenitis scores reported in this study were very low, the average score was less than 2, using a scale of 0 (no lesions noted) to 10 (severe ulcerative), according to Bigham and McManus (28). Also, cattle fed T1 and T5 presented larger MPA, which may have contributed to a larger development of rumen epithelium, which allows a faster SCFA clearance; however, larger MPA in the current study did not influence ASA. The rumen wall absorptive surface area (ASA) is the morphometric variable most correlated to the speed of SCFA absorption, playing an important role to increase the ruminal pH to adequate levels (44). Rigueiro et al. (9) observed that the shortening of the adaptation period for cattle fed only VM did not negatively impact rumenitis score; however, cattle receiving VM by 14 days presented a higher incidence of rumen lesions when compared to those fed MON+VM by 14 days (0.85 vs. 0.38, respectively). In addition, the authors reported that cattle fed virginiamycin for 9 days had lower rumen development in the number of papillae, mean papillae area, ASA and papillae area expressed as % of ASA. Moreover, there is evidence that VM supplementation results in increased propionate synthesis and reduced acetate and butyrate concentration, as well as reduced lactate production and increased ruminal pH (45). In this context, decreasing the ruminal acetate/propionate ratio may have contributed to a development of rumen epithelium, increasing the SCFA absorption. Squizatti et al. (46) observed a quadratic effect for adaptation length when only VM for mean pH, duration of pH below 5.2 and 6.2, where cattle consuming VM adapted for 9 days had higher mean pH and shorter period of pH below 5.2 and 6.2 compared to animals adapted in 6 days. However, it is important to note that these results do not guarantee that adaptation length can be reduced since it was observed lower DMI for cattle adapted for 9 days, as already described by Rigueiro et al. (9), a fact associated with inadequate adaptation. On other hand, the authors reported that animals consuming only VM and adapted for 14 days had higher maximum pH and acetate:proprionate ratio, as well as lower ox-redox potential than cattle receiving MON+VM for 14 days.

## 4. Conclusion

In conclusion, the withdrawal of MON when associated with VM during the last 40 days of the feedlot period did not increase DMI; however, this withdrawal of MON combined with a higher energy diet during the final third of the feedlot period improved overall final BW, ADG and HCW of Nellore cattle when compared to bulls fed either MON or VM, but did not positively impact feedlot performance and carcass characteristics when compared to cattle that had MON withdrawn by the end of the adaptation period. According to the results, Nellore cattle should be fed high-concentrate diets containing MON and VM during adaptation, and only VM during the finishing period to improve overall feedlot performance.

## Data availability statement

The raw data supporting the conclusions of this article will be made available by the authors, without undue reservation.

## Ethics statement

The animal study was reviewed and approved by São Paulo State University Ethical Committee for Animal Research (Protocol No.: CEUA 154/2016).

## Author contributions

AR and DM: conceived and designed study, collected and complied, and analyzed data. MP, AS, AP, LF, ED, BD, DE, JD, KS, LS, and AN: investigation. AR, JS, and DM: provided intellectual input and drafted and edited manuscript. All authors contributed to the article and approved the submitted version.
